# Photoacoustic Wavefront Shaping with High Signal to Noise Ratio for Light Focusing Through Scattering Media

**DOI:** 10.1038/s41598-019-40919-6

**Published:** 2019-03-13

**Authors:** Jialin Sun, Bin Zhang, Qi Feng, Huimei He, Yingchun Ding, Qiang Liu

**Affiliations:** 1 Department of Physics, Beijing University of Chemical Technology, Beijing, 100029 China; 2 State Key Laboratory of Precision Measurement Technology and Instruments, Department of Precision Instruments, Tsinghua University, Beijing, 100084 China

## Abstract

Noninvasive light focusing and imaging through a scattering medium can be achieved by wavefront shaping using the photoacoustic signal as feedback. Unfortunately, the signal to noise ratio (SNR) of the traditional photoacoustic method is very low, which limits the wavefront shaping focusing speed and intensity. In this paper, we propose a completely new photoacoustic-signal-extraction method which combines wavelet denoising and correlation detection. With this method, the SNR of the photoacoustic signal reaches 25.2, 6.5 times higher than that of the unprocessed photoacoustic signal. Moreover, we achieve the simultaneous multipoint focusing, which is crucial for improving the speed of scanning imaging. The superior performance of the proposed method was experimentally demonstrated in extracting and denoising the photoacoustic signals deeply buried in noise, one critical step in *in vivo* photoacoustic imaging.

## Introduction

Light focusing and imaging through highly scattering biological tissues is of great interest for biomedical applications. Traditional focusing and imaging techniques, such as optical coherence tomography (OCT) and confocal laser scanning microscope, utilize ballistic light. Since the ballistic light decreases exponentially with the penetration depth, these techniques can only be applied to superficial biological tissues^1–3^.

The emergence of wavefront shaping technology makes it possible to utilize diffuse light to focus and image, which effectively improves the depth of light focusing and imaging^4^. By compensating for the wavefront of the light before it arrives at scattering media, the light passing through scattering media can be controlled. Optical wavefront shaping technology has made great progress. It can now achieve ~*μs* level focusing speed and ~*μm* level resolution^5,6^. However, most of the researches so far have only focused light behind the scattering medium with pure optical wavefront shaping method. Because a feedback signal at the target point is needed during the wavefront shaping. Usually the light intensity at the target point acquired by the charge coupled device (CCD) camera is used as the feedback signal^7,8^. Since the CCD camera cannot be placed inside biological tissue without any damage, it is fatal for noninvasively wavefront shaping in living tissues. Some recent techniques have achieved noninvasive light focusing based on optical and acoustic interactions such as acousto-optic effect and photoacoustic effect^9,10^. The time-reversed ultrasonically encoded (TRUE) optical focusing technique utilizes the acousto-optic effect to generate an ultrasonic guide star, and the ultrasonically encoded light was used as the feedback signal. TRUE uses holography to time-reverse the ultrasonically encoded light and then forms a focal point within the scattering medium^11^. However, this method requires complex experimental optical setup and tedious experimental procedure. Besides, the ultrasound encoded light accounts for a fairly low proportion of the total scattered light which makes the signal detection more difficult^12^.

Photoacoustic wavefront shaping can overcome these shortcomings. Instead of optical signals, it uses acoustic signals generated based on photoacoustic effect as the feedback signals. The photoacoustic effect refers to the phenomenon that the absorber absorbs the energy of the laser pulse to generate thermal expansion and ultrasonic waves^13^. Comparing to the light, but the scattering of ultrasonic waves in biological tissues is much weaker. Photoacoustic wavefront shaping not only provides noninvasive feedback signals, but also improves the depth of light focusing and imaging. However, the application of photoacoustic signals to wavefront shaping is still relatively lacking. T. Chaigne *et al*. used the photoacoustic effect to measure the photoacoustic transmission matrix and realized photoacoustic wavefront shaping. Finally, they got an approximately 6 times enhancement of the photoacoustic signal^14^. Most researches of photoacoustic wavefront shaping use the phase-only spatial light modulator (SLM) to control the wavefront of incident light, but its application in living tissues with short decorrelation time is difficult considering the refresh rate of SLM (100 Hz)^14–16^. L. V. Wang *et al*. introduced a method that utilizes a digital micromirror device (DMD), which is capable of operating at 22 kHz,in the photoacoustic wavefront shaping system to increase the focusing speed^17^. However, these methods are limited by the problem that the SNR of the photoacoustic signal is poor. DMD surface can endure lower pulsed laser fluence (<200 *μJ*/*cm*^2^) than that of SLM (<40 *mJ*/*cm*^2^). Lower pulse laser fluence means weaker photoacoustic signal and lower SNR. The commom method to improve the SNR is to collect and average the photoacoustic signals for many times. This method can only slightly improve the SNR but takes much more time. L. V. Wang *et al*. averaged 64 acquisitions of photoacoustic signals, raising the SNR to 3.9. The optimization process still took about 2 hours with a high repetition rate pulse laser (1 kHz). In addition, O. Tzang *et al*. proposed a lock-in detection method and for photoacoustic signals and improved the SNR by an order of magnitude. However, their methods require complicated signal processing circuits and expensive light modulation devices such as acousto-optic modulator. Their experiments used a pulsed laser with a repetition rate of 20 kHz. It took 6 minutes to optimize the linear photoacoustic signals and nearly one hour for the nonlinear photoacoustic signals. The photoacoustic signals was enhanced 9 times and 16 times, respectively.

In this paper, we propose a method that combines wavelet denoising and correlation detection to improve the SNR of the photoacoustic signals. The acquired photoacoustic signals are digitallly processed and there is no need of any additional signal processing circuit. The SNR of the photoacoustic signal reaches 25.2, which is about 6.5 times higher than that of the photoacoustic signal before denoising. The proposed method significantly improves the focusing speed and intensity of the wavefront shaping technology using photoacoustic signals as feedback. With the improvement of SNR, we enhance the intensity of single point photoacoustic signal by 7.83 times using a binary amplitude modulation device DMD. Further, we achieve simultaneous multipoint focusing with a low frequency ultrasonic transducer. We can still obtain relatively high enhancements (3.7 and 2.4 times, respectively) when the two points average the energy of light. This is extremely advantageous for improving the speed of scanning imaging after focusing. Since a large number of repeated acquisitions of photoacoustic signals are not required, we achieve faster focusing speeds with lower pulse repetition rates (10 Hz).

## Method

In this paper, we combine wavelet denoising and correlation detection to detect photoacoustic signals deeply buried in noise with improved SNR.

### Wavelet denoising

Wavelet denoising has been widely used in signal and image processing technology for many years^18–20^. Different from simple frequency domain filtering, it filters out noise while preserving the details of the useful signal as much as possible. Wavelet denoising processes wavelet transform coefficients of signals and noiseis based on different representations of wavelet transforms at different scales of signal and noise^21^. The essence of denoising is to reduce or even eliminate the wavelet coefficients introduced by noise, while maximizing the wavelet coefficients of the signals. After wavelet transform, the wavelet coefficients of the signals have a strong correlation at each scale, while the wavelet coefficients corresponding to the noise have no such obvious correlation between these scales.

Ignoring the attenuation of sound waves, the basic model of noise-inducing photoacoustic signals can be written as follows^22^:

1 $$h=s(r)+\sigma \omega $$

where *ω* is standard Gaussian white noise, which follows *ω*~*N*(0, 1) and *σ* is the noise level. *h* is the signal received by the oscilloscope. *s*(*r*) is the photoacoustic signal at *r* in the medium. The signal at position *r* can be written as follows^13^:

2 $$s(r)={\rm{\Gamma }}{\mu }_{a}(r)\phi (r)$$

Γ is the Grüneisen parameter which is related to the properties of the medium, *μ*_*a*_(*r*) is the absorption coefficient at *r* in the medium, *φ*(*r*) is the light fluence at position *r*. The detected photoacoustic signals are also related to the response of the detector. Since our experiment used the same ultrasonic transducer, this problem was not considered. The purpose of the wavelet denoising is to suppress the noise part *σω* of *h* and to recover *s*(*r*).

On the choice of the mother wavelet, one method is to select a wavelet similar to the waveform of the signal to be denoised as the denoising effect of the mother wavelet is better^23^. The waveform of the photoacoustic signal is related to the size of the absorber, the frequency of the ultrasonic transducer, and the relative position of the ultrasonic transducer and the absorber^24^. In this experiment, the daubechies wavelet, the symlet wavelet and the coiflet are tested. They are all similar to the waveform we have acquired and they are more commonly used in one-dimensional signal denoising. Since the smoothness of the signal affects the accuracy of the correlation detection method, we finally chose the fourth-order daubechies wavelet as the mother wavelet considering the smoothness and denoising. And the level of decomposition is eight. The threshold of wavelet denoising calculated using the common threshold proposed by Donoho and Johnstone for VisuShrink, which is $K=\sigma \sqrt{2\,{log}\,M}$ where M is the signal length and *σ* is the estimated value of noisy variance. For one-dimensional signal the threshold results in an estimate asymptotically optimal in the minimax sense (minimizing the maximum error over all possible - sample signals)^25^.

### Correlation detection

**Figure 1:**
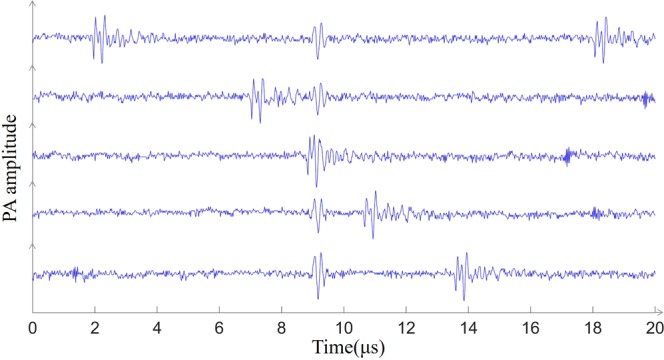
Mixing of photoacoustic signals and clutters. The five waveforms arranged vertically represent waveforms acquired by the oscilloscope at different times. The position of the photoacoustic signal is invariable, and the clutters appear at different positions at each acquisition and even mix with the photoacoustic signal, making the photoacoustic signal difficult to obtain accurately.

In addition to the photoacoustic signal and Gaussian white noise, the signal detected by the oscilloscope during the experiment was also mixed with clutters (Fig. 1) caused by device’s electrostatic and amplifier noise.

**Figure 2:**
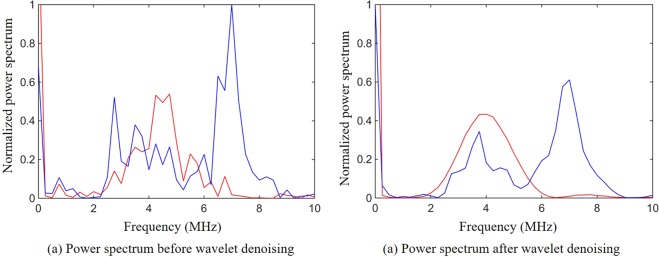
Spectral analysis of signals and clutters. (**a**) The power spectrum before wavelet denoising where the red line represents the power spectrum of the signals and the blue line represents the power spectrum of the clutters. (**b**) The power spectrum after wavelet denoising where the red line represents the power spectrum of the signals and the blue line represents the power spectrum of the clutters.

We performed a spectrum analysis of the signals and clutters before and after wavelet denoising (Fig. 2). From the spectrum analysis we can clearly see that these clutters usually contain the same frequency components as the photoacoustic signals (Fig. 2). This means band-pass filtering cannot eliminate these clutters which have a great influence on the extraction and optimization of the feedback signals.

In this paper, we propose a method that use the correlation coefficient to distinguish between photoacoustic signal and noise. The normalized correlation coefficient is calculated as equation^26^:

3 $${\gamma }_{xy}=\frac{{\sum }_{n=0}^{N-1}\,[x(n)-\bar{x}]\,[y(n)-\bar{y}]}{\sqrt{{\sum }_{n=0}^{N-1}\,{[x(n)-\bar{x}]}^{2}\,{\sum }_{n=0}^{N-1}\,{[y(n)-\bar{y}]}^{2}}}$$

where *γ*_*xy*_ is a normalized correlation coefficient, *N* is the number of template points, *x*(*n*) are the template points, *y*(*n*) are the signal points under analysis, $\bar{x}$ is the mean of the template points, $\bar{y}$ is the mean of the signal points. The value of the normalized correlation coefficient is between −1 and 1, and 1 denotes the exact match between the signal under test and the template signal.

Although the speckle changes with the random-amplitude mask loaded on the DMD, the waveform of the photoacoustic signal changes very little since the same ultrasonic transducer is used. Thus, we could use the photoacoustic signals as templates to distinguish different waveforms of the photoacoustic signals and the noises. The selection of photoacoustic signal templates is described in detail in the later section of this paper.

In our experiments, different waveforms of the photoacoustic signals and the noises were collected, and their correlation coefficients were calculated. The correlation coefficients for the photoacoustic signals corresponding to different masks are above 0.8, and the correlation coefficients between photoacoustic signals and noises are below 0.4. Therefore, we set the correlation coefficient threshold to 0.7. After the correlation coefficient was calculated, the waveform with a correlation coefficient higher than 0.7 was selected as the photoacoustic signal, and the signal with the correlation coefficient lower than 0.7 was ignored. The photoacoustic signals corresponding to the same mask were collected five times for averaging.

**Figure 3:**
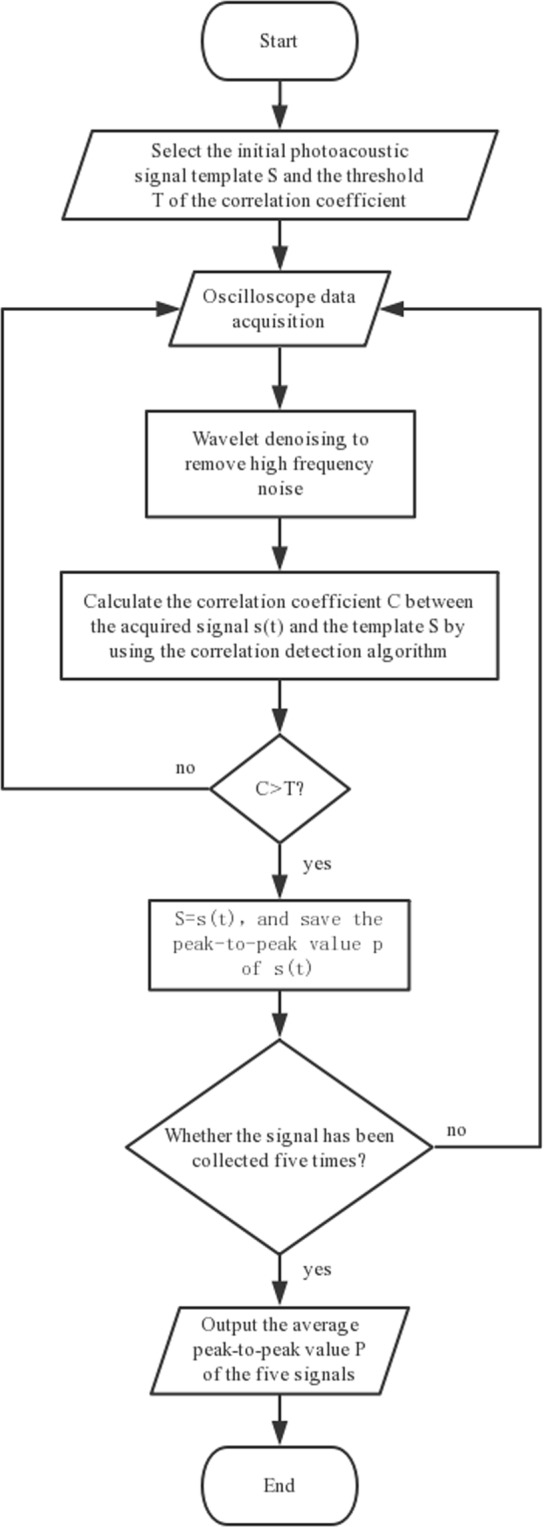
Flow chart of combined method for wavelet denoising and correlation detection.

During the experiment, we perform wavelet denoising on each acquired signal and use correlation detection to judge whether the collected signals are photoacoustic signals. The flow chart of the method is shown in Fig. 3.

The algorithm can be roughly divided into the following steps as shown in Fig. 3. (1) Once the program starts, the template S of the initial photoacoustic signal and the threshold T of the correlation detection algorithm are input. The template of the initial photoacoustic signal can be obtained through experimental measurement with the DMD pixel fully open. (2) The signal s(t) is acquired by the oscilloscope. (3) The acquired signal is denoised by wavelet and then the correlation coefficient C is calculated with the initial template. When the correlation coefficient C is lower than the set threshold T, it continues to return to step 2. (4) When the correlation coefficient C is higher than the set threshold T, the initial template S is replaced with the signal s(t) and save the peak-to-peak value p of s(t). Since the amplitude of the signal increases with the increase of generation during the optimization process, the replacement of the template further improves the accuracy of the relevant detection method. Each DMD mask collects five times signals, and then outputs the average peak-to-peak value P of the photoacoustic signals as the evaluation function of the genetic algorithm. Through the combination of the wavelet denoising and correlation detection, we can not only improve the SNR of photoacoustic signals, but also effectively filter out clutters. Even though the waveforms produced by different absorbers are different, we can focus multiple objects (such as blood vessels and muscles) by selecting multiple templates. Moreover, when there are different targets in the focusing range that can generate different photoacoustic signals, selecting an appropriate template can focus on different targets.

### Experimental setup

**Figure 4:**
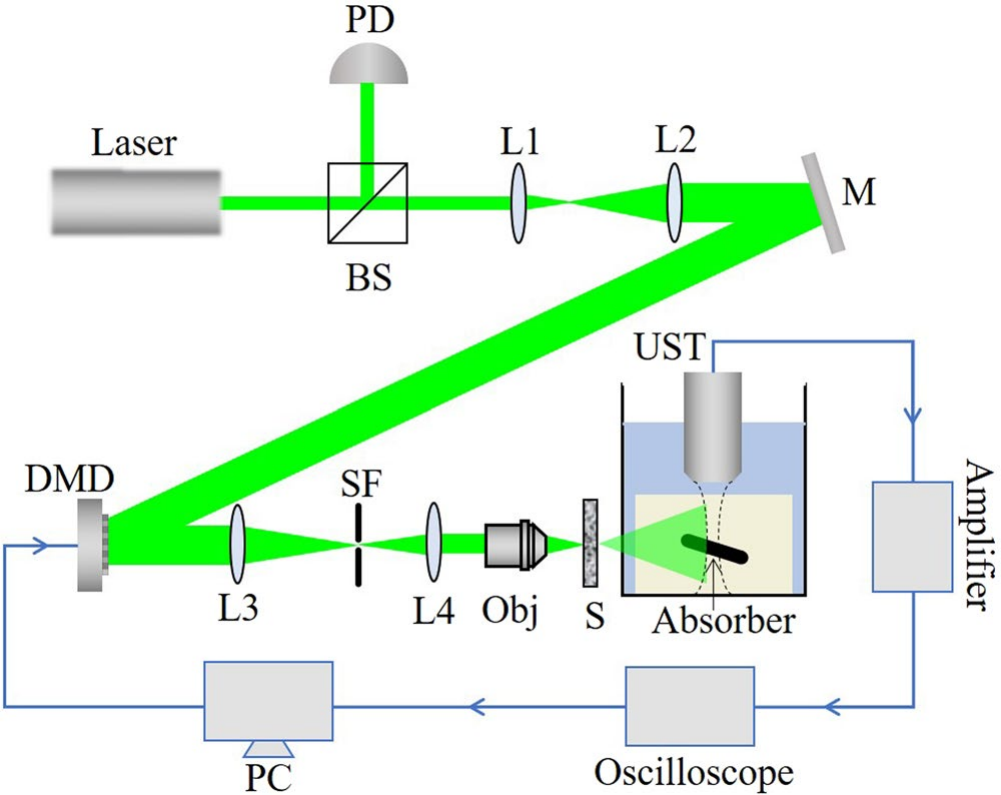
Experimental setup. BS: Beam splitter; PD: Photodiode; L1, L2, L3, L4: Lens; M: Mirror; SF: Spatial filter; Obj: Objective; S: Sample; UST: Ultrasound transducer; The absorber is embedded in an agarose gel and placed in a water tank. The absorber is perpendicular to both the laser irradiation direction and the axial direction of the ultrasonic transducer. The blue line in the figure denotes the circuit part in the experiment.

The experimental setup is shown in Fig. 4. We used a 532 *nm* pulsed laser (SGR-10, LABest) with a repetition rate of 10 Hz and a pulse energy of ~800 *μJ*. The laser beam was expanded and collimated, then it was reflected by a mirror onto the DMD (DLP6500, Texas Instruments). The light reflected by the DMD was constricted and filtered by a 4*f* system and then focused by a 10× objective (NA = 0.25) onto a scattering diffuser (Diffuser 83419 Edmund Optics). A portion of the beam was split into the photodiode (S5971, Hamamatsu Photonics) to compensate for the energy fluctuations of the pulse laser. The photoacoustic signals were generated from an absorber embedded in an agarose gel. The absorber used in this paper is a 150 *μm* diameter black nylon thread, and the nylon thread was placed 3 cm behind the scattering sample to produce speckles approximate to the absorber. A 5 MHz ultrasonic transducer (5Z10SJ30DJ, SIUI) was used to collect the photoacoustic signals. The center frequencies of the photoacoustic signals generated by absorbers of different sizes are different. The center frequency is given by *f* ≈ 0.66*c*_*s*_/*D*_*a*_, Where *c*_*s*_ is the speed of sound in water and *D*_*a*_ is the diameter of the absorber^24^. The center frequency of the generated signals should be 6.6 MHz. Thus we use a 5 MHz ultrasonic transducer, whose bandwidth can cover the center frequency of the photoacoustic signals generated by our absorber. The photoacoustic signals were amplified by an amplifier (ZFL-500LN+, Mini-Circuits) and then acquired by an oscilloscope (WaveMaster 806Zi-A, LeCroy). The computer dealt with the data collected by the oscilloscope in real time and refreshed the mask loaded on the DMD according to the photoacoustic feedback signal. After the focusing experiment was complete, we replaced the absorber and the water tank with a CCD to obtain an image of the focus (MER-031-300GM, Daheng Imavision).

## Results

In the experiment, we first implemented a wavefront shaping light focusing experimentin which the energy of incident light is relatively low and the corresponding photoacoustic signal used as the feedback has a low SNR. Then, we experimentally proved the effectiveness of wavelet denoising and correlation detection method by improving the optimization results. For the optimization,we implemented a geneti algorithm (GA) with stronger roubustness^27^.

In the optimization process, we divided the DMD into individual segments, each segment contains micromirrors. The larger the segment size, the larger the speckle after the laser passes through the scatterer, and the more it matches our larger absorption region, but larger segment size reduces the degrees of freedom we can control. Considering both aspects of speckle size and degrees of freedom, we finally chose the above segment size. Photoacoustic signals for different DMD masks were collected five times for averaging. The optimization process used a genetic algorithm which used photoacoustic signals as feedback. Random masks were generated as the initial populations, which were ranked in descending order by the fitness of the initial populations. In this experiment, the fitness value is the peak-to-peak value of the photoacoustic signal. Masks with high fitness values in each generation are easier to be selected to generate next generation by crossing and mutating. Through multiple iterations, an optimal mask, i.e. the mask with the largest peak-to-peak value of photoacoustic signal at the target point, was obtained^28^. In the experiment, we used 80 generations, and each generation contains 50 populations.

By calculating the ratio of the amplitude of the photoacoustic signal to the standard deviation of the noise without laser irradiation, we can measure the SNR^17^. The wavelet denoising can effectively improve the SNR of the photoacoustic feedback signal.

**Figure 5:**
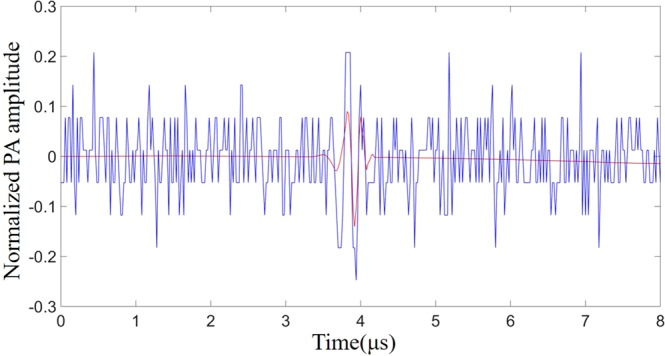
The effect of wavelet denoising. The blue line represents the photoacoustic signal acquired by the oscilloscope before wavelet denoising. The red line represents the photoacoustic signal after wavelet denoising.

We calculated the SNR for 100 randomly acquired photoacoustic signals with different random masks loaded on the DMD. When the random mask was loaded, the average SNR of the photoacoustic signals without wavelet denoising is 3.86. The SNR of the photoacoustic signal reaches 25.2, which is about 6.5 times higher than that of the photoacoustic signal before denoising. The reason for the low initial SNR is that in addition to white noise, there is quantization noise introduced by oscilloscope in process of converting analog signals to digital signals. Figure 5 shows a representative result of increase of SNR of the photoacoustic signal processed by wavelet denoising. And the signals without clutters were selected by the correlation detection method.

In theory, the enhancement of the photoacoustic signal of optimal mask compared to the photoacoustic signal of random mask can be calculated by the following formula^29^:

4 $$\eta =\frac{1/2+(N-1)/2\pi }{M}\approx \frac{N}{2\pi M}$$

where *M* is the number of independent optical modes contained within the absorbing area of the absorber inside the acoustic focal region, and *N* is the number of controllable modes on the DMD. Ultrasound transducer focus diameter $\varphi ={c}_{s}F/f\,D$, which is 880 *μm* in our experiment. *c*_*s*_ is the speed of sound. *F* is the focal length of the ultrasonic transducer. *f* is the frequency of the ultrasonic transducer. *D* is the crystal diameter of the ultrasonic transducer. The absorber is a 150 *μm* diameter black nylon thread. Therefore, the size of the absorbing area inside the focal region of the transducer is 150 *μm* × 880 *μm* = 132000 *μm*^2^. According to the speckle diameter (150 *μm*) at the target position, we can calculate $M=132000\,\mu {m}^{2}$/$[\pi \cdot {(75\mu m)}^{2}]\approx 7.47$. Therefore, the theoretical enhancement $\eta =(32\times 18)$/$(2\times \pi \times 7.47)\approx 12.27$.

**Figure 6:**
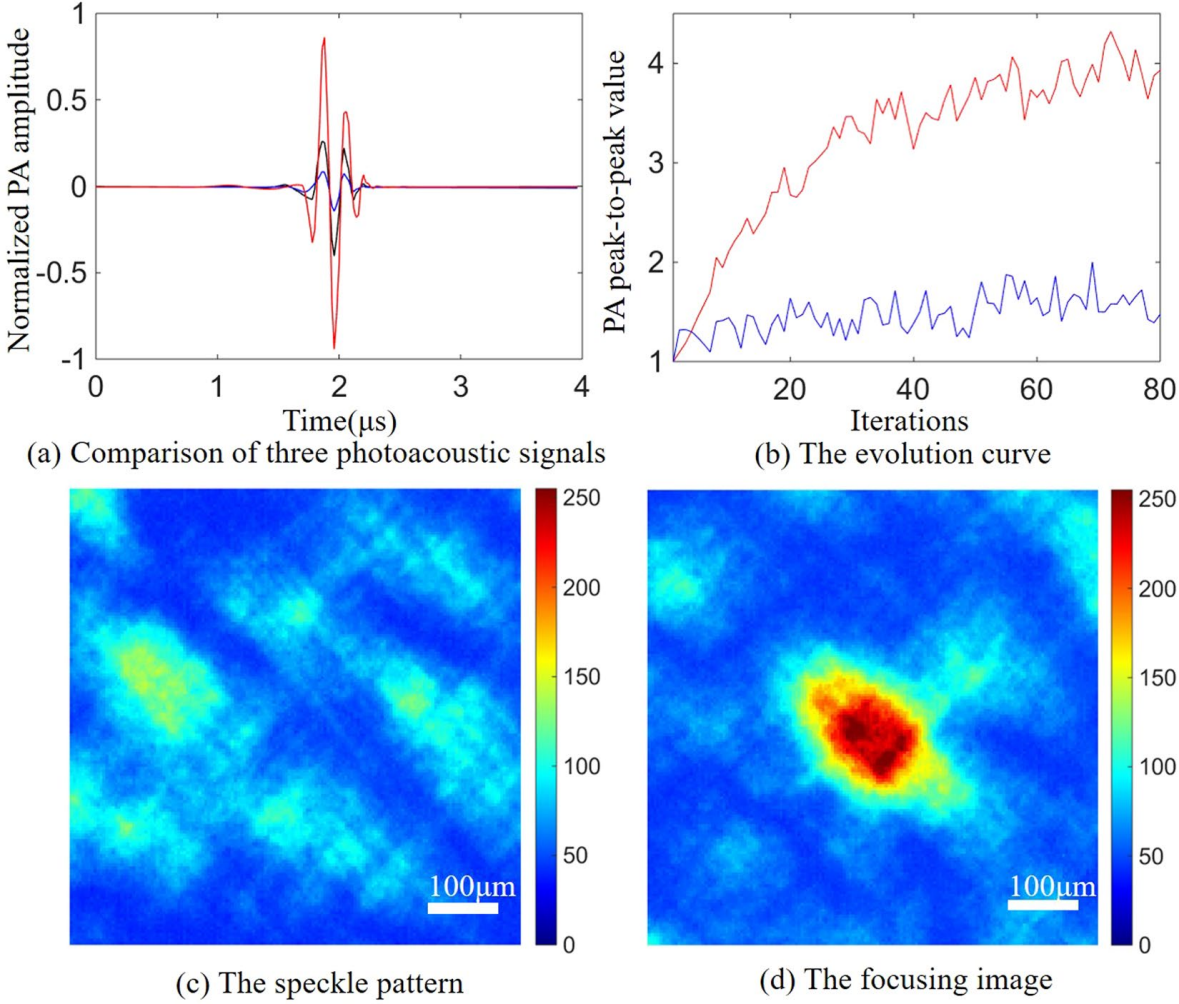
Single-point focusing experiment. (**a**) Comparison of three photoacoustic signals. The blue line represents the corresponding photoacoustic signal when the DMD loads a random mask, and the black line represents the corresponding photoacoustic signal when the DMD pixels are fully turned on. The red line represents the photoacoustic signal of the optimal mask after optimization. (**b**) The evolution curves of the averaged fitness value in the evolution processes of the genetic algorithm. The red line indicates the result using wavelet denoising and correlation detection method, and the blue line indicates the signals only averaged. (**c**) The speckle pattern captured by the CCD before optimization. (**d**) The focusing image captured by the CCD after optimization.

**Figure 7:**
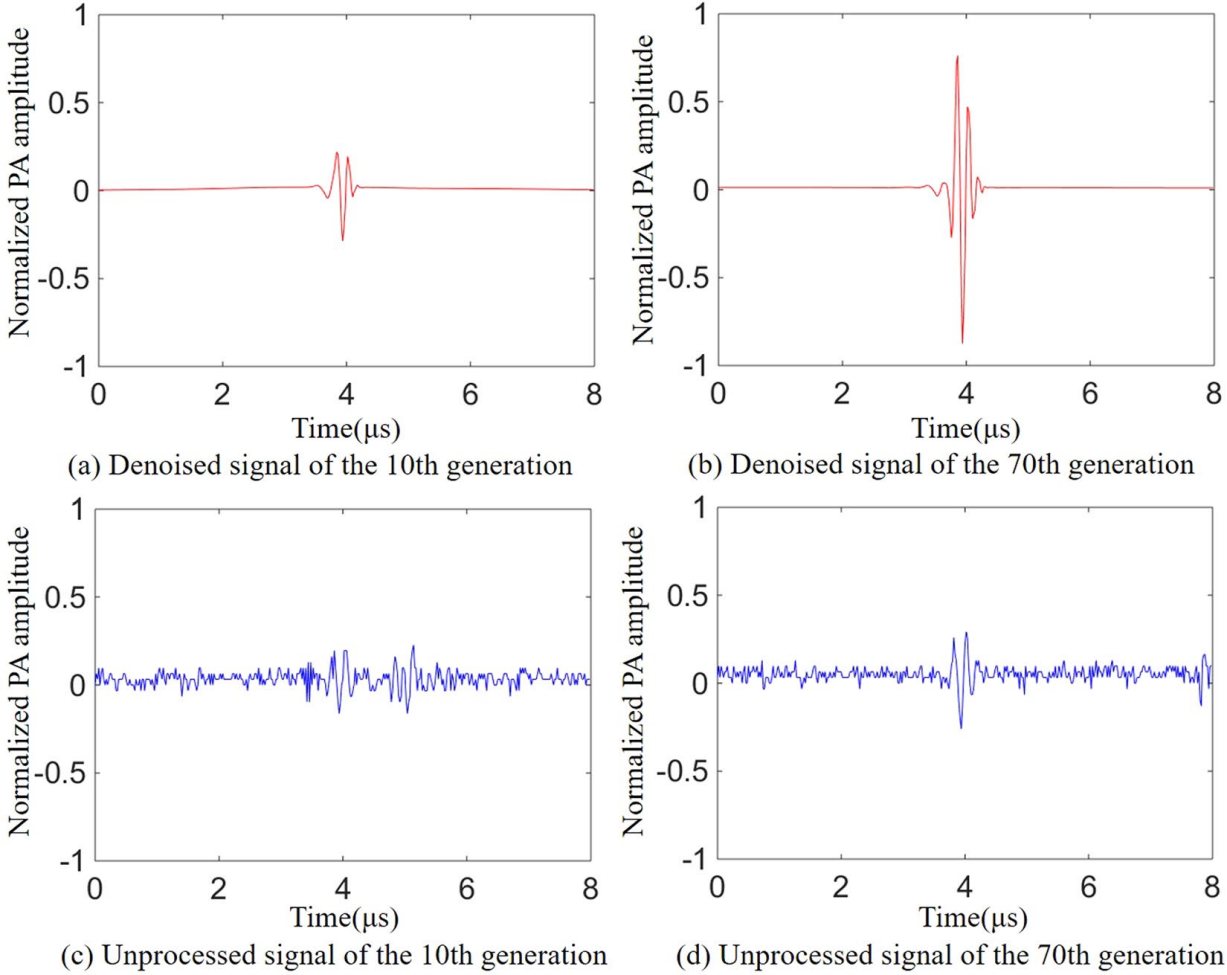
The photoacoustic signals during optimization. (**a**,**b**) Respectively represent the photoacoustic signals of the 10th and 70th generations which were obtained by wavelet denoising and correlation detection. (**c**,**d**) Denote the photoacoustic signals of the 10th and 70th generations which were obtained without wavelet denoising and correlation detection.

The optimized result of the photoacoustic signal after optimization is shown in Fig. 6(a), The photoacoustic signal after optimization is approximately 7.83 times higher than the photoacoustic signal obtained with random mask loaded on the DMD, and 2.71 times higher than the photoacoustic signal obtained when all the DMD pixels are open. Although only half of the DMD pixels are turned on in the optimal mask (i.e. only half of the intensity of the incident light reached on the scattering medium), the photoacoustic signal generated by the optimal mask is still stronger than the photoacoustic signal when all the DMD pixels are opened. The main cause is that the optimal mask selected the segments which added constructively at the target point^30^. The enhancement (7.83) obtained in our experiments is very close to the theoretical enhancement (12.27), owing to our high signal-to-noise ratio photoacoustic signals. Figure 6(b) shows the evolution curve of the averaged fitness value of each generation in the optimization process. The red line represents the experimental results obtain by the method we proposed which combines the wavelet denoising with the correlation detection, while the blue line indicates that only the data averaging method is used to improve the SNR. Figure 6(c,d) show the speckle patterns captured by the CCD before and after optimization during the optimization process of Fig. 6(a). We present the waveforms of two typical generations in two evolutionary processes (Fig. 7). As expected, due to the lower SNR and the influence of clutters, the enhancements obtained with the averaging method are unsatisfactory.

During the experiment, we noticed that although the waveforms of the photoacoustic signals before and after the optimization are similar, there are still some differences between different generations. This may have a negative effect on our correlation detection method. Therefore, we used the photoacoustic signal obtained during each iteration as the correlation detection template for the next acquisition of the photoacoustic signal. This maximizes the assurance that each time what participates in the iteration is the photoacoustic signal received by the ultrasonic transducer instead of the noise.

Figure 7(a,b) show the photoacoustic signals acquired at the 10th and 70th generations in the evolutionary process shown in Fig. 6 (red line) that used wavelet denoising and correlation detection method. Figure 7(c,d) show the photoacoustic signals acquired at the 10th and 70th generations of the evolutionary process shown in Fig. 6(b) (blue line) that without wavelet denoising and correlation detection. As shown in Fig. 7, when the wavelet denoising and correlation detection methods is not used, the enhancement of the photoacoustic signal is relatively low due to the poor SNR and clutters. The wavelet denoising and correlation detection methods proposed by us not only improves the SNR of the photoacoustic signal but also effectively filters out the clutters.

**Figure 8:**
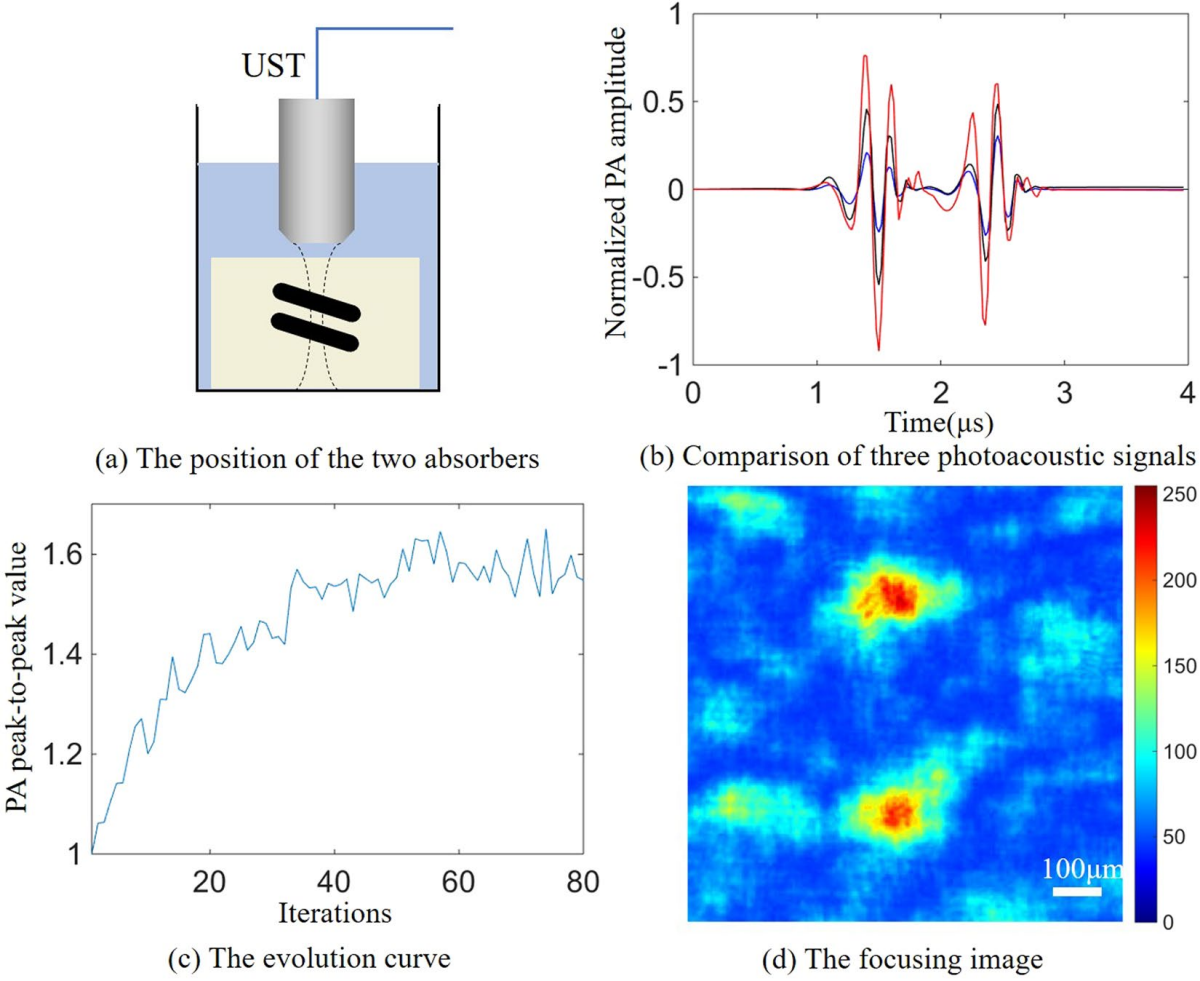
Two-point focusing experiment. (**a**) The position of the two absorbers, where both absorbers are perpendicular to the laser incident direction and the axial direction of the ultrasonic transducer. (**b**) Comparison of three photoacoustic signals. The blue line represents the photoacoustic signal when the DMD loads a random mask, and the black line represents the photoacoustic signal when the DMD pixels are fully turned on. The red line represents the photoacoustic signal under the optimal mask after optimization. (**c**) The evolution curve of the averaged fitness value (in the two-point focusing experiment, the fitness value is the square root of the two photoacoustic signals’ product of peak to peak value) in the evolution process of the genetic algorithm. (**d**) The focusing image captured by the CCD after optimization.

We have further actualized two-point focusing experiment with two absorbers. Since the photoacoustic signals from different target points reaches the transducer at different times and our ultrasonic transducers have a large focal region, we can simultaneously receive the photoacoustic signals from multiple absorbers. Figure 8(a) shows a phantom containing two target absorbers. Both absorbers are made of the 150 *μm* diameter black nylon thread and embedded in an agarose gel. The absorber was placed 3 cm behind the scattering sample, and the two absorbers are separated by 500 *μm*. As in the case of single absorber, the pulsed laser first passed through the scattering medium – ground glass before it irradiated onto the agarose gel.

Figure 8 shows that two-point focusing results in lower signal enhancement due to the enlargement under the absorption area. The photoacoustic signals of the optimal masks for the two absorbers were respectively 3.7 and 2.4 times stronger than the photoacoustic signals under the random masks. Theoretically, as shown in Eq. 4, the enhancement of photoacoustic signal for two-point focusing experiment should be half of the single-point focusing enhancement, i.e. 6.135. The difference in the enhancement of the photoacoustic signals for the two absorbers is mainly due to the sensitivity of the ultrasonic transducer varies in the focal region^16^.

## Discussion and Conclusions

In the experiment we used an ultrasonic transducer with a low center frequency (5 MHz). Lower frequency result in a larger ultrasound focal region, which increases the number of speckle patterns within the ultrasound focal region. Then, the enhancement of the photoacoustic signal becomes lower. However, large ultrasound focal region allows us to obtain the feedback signals of multiple absorbers. In addition, lower frequency photoacoustic waves experience less attenuation in living tissues, which is of great significance in further increasing light focusing depth with photoacoustic method^13^.

The application of DMD makes it possible to achieve fast light focusing by photoacoustic wavefront shaping. However, for the photoacoustic wavefront shaping, the DMD can withstand low-energy pulse laser which further reduces the SNR of the photoacoustic signals. The method we proposed in this paper which combines wavelet denoising and correlation detection can effectively improve the SNR of the photoacoustic signals and has no need to acquisition of a large number of photoacoustic signals for averaging.

The overall focusing speed depends on the acquisition time of a single photoacoustic signal and the total duration of the optimization^15^. The single photoacoustic signal processing time of the proposed method is about 50 *ms*, which is mainly limited by the computing speed of the computer. Therefore, with a better performing computer or FPGA, the processing time can be shortened andbecomes more promising for living tissues with short decorrelation time^29^. Our focusing experiments takes 30 minutes, which are mainly limited by the repetition rate of the pulsed laser (10 Hz). These are not fundamental restrictions. Faster photoacoustic wavefront shaping can be achieved with our method using a higher repetition rate pulsed laser and a higher speed calculator.

We propose a method to enhance the SNR of the photoacoustic signals in photoacoustic wavefront shaping, which can be used to extract and denoise photoacoustic signals deeply buried in noise. Compared with previous work, our method focus quickly (30 minutes) using a low repetition rate pulse laser. Our method is simple and does not require any additional expensive experimental equipment or complicated signal processing circuits. The experiments demonstrated that our method increased the SNR of the photoacoustic signals from the average value 3.86 to 25.2, which increases nearly 6.5 times. High SNR of the photoacoustic feedback signals can not only improve the focusing speed of the wavefront shaping, but also improve the intensity of the focus. Using a binary amplitude modulation device (DMD), we achieved relatively high photoacoustic signal enhancement (7.83 times). In addition, benefit from improved SNR, we achieved simultaneous multipoint focusing with low frequency ultrasound transducer, and the two photoacoustic signals enhanced 3.7 and 2.4 times respectively.

The proposed method can effectively identify the photoacoustic signals from white noise and clutters, but when the white noise is too serious (*SNR* ≈ 1), the signal is completely submerged by noise, and wavelet denoising becomes ineffective. In this case, the method that extract the signal from the white noise first with the frequency domain filtering method and then eliminate the influence of the clutters which overlap with the photoacoustic signal in the frequency domain with the correlation detection algorithm may be feasible.

In order to improve the optimization result, we selected the speckle grain similar to the size of the absorber. In biological tissues, we are unable to control the speckle grains size. Smaller speckle grains size will result in an increase in the number of speckle grains that are irradiated onto the absorber. This requires more DMD blocks to increase the controllable degrees of freedom, or the optimization effect will go unsatisfactory. However, higher controllable degrees of freedom mean longer optimization time. As the speckle size becomes smaller, it may occur that a multiple of optimized speckles appear in the area where the ultrasonic focusing region overlaps with the absorber. This problem can be solved using the nonlinear photoacoustic effect^31^. Wavefront shaping methods based on non-linear photoacoustic effect have no requirement for speckle size. Due to the non-linear response of the absorber, a focus containing only one speckle grain will be formed in the ultrasound focal region after optimization. The signal acquisition and detection method proposed in this paper is equally effective for nonlinear photoacoustic wavefront shaping.

The method we proposed has great advantages in the case of low SNR photoacoustic signals. It can effectively extract and denoise the photoacoustic signals and is widely applicable to general photoacoustic imaging and photoacoustic wavefront shaping. The use of genetic algorithm further enhances the robustness of the method, which is extremely important for its application in living tissues.
